# Effect of visual distraction and auditory feedback on patient effort during robot-assisted movement training after stroke

**DOI:** 10.1186/1743-0003-8-21

**Published:** 2011-04-23

**Authors:** Riccardo Secoli, Marie-Helene Milot, Giulio Rosati, David J Reinkensmeyer

**Affiliations:** 1 Robotics Lab, Department of Innovation in Mechanics and Management, University of Padua, Via Venezia 1, 35131 Padova, Italy; 2 Biomechatronic Lab., Departments of Mechanical and Aerospace Engineering, University of California, 4200 Engineering Gateway, Irvine, CA 92697-3875 Irvine, USA; 3 Departments of Mechanical and Aerospace Engineering, Anatomy and Neurobiology, and Biomedical Engineering, University of California, 4200 Engineering Gateway, Irvine, CA 92697-3875 Irvine, USA

## Abstract

**Background:**

Practicing arm and gait movements with robotic assistance after neurologic injury can help patients improve their movement ability, but patients sometimes reduce their effort during training in response to the assistance. Reduced effort has been hypothesized to diminish clinical outcomes of robotic training. To better understand patient slacking, we studied the role of visual distraction and auditory feedback in modulating patient effort during a common robot-assisted tracking task.

**Methods:**

Fourteen participants with chronic left hemiparesis from stroke, five control participants with chronic right hemiparesis and fourteen non-impaired healthy control participants, tracked a visual target with their arms while receiving adaptive assistance from a robotic arm exoskeleton. We compared four practice conditions: the baseline tracking task alone; tracking while also performing a visual distracter task; tracking with the visual distracter and sound feedback; and tracking with sound feedback. For the distracter task, symbols were randomly displayed in the corners of the computer screen, and the participants were instructed to click a mouse button when a target symbol appeared. The sound feedback consisted of a repeating beep, with the frequency of repetition made to increase with increasing tracking error.

**Results:**

Participants with stroke halved their effort and doubled their tracking error when performing the visual distracter task with their left hemiparetic arm. With sound feedback, however, these participants increased their effort and decreased their tracking error close to their baseline levels, while also performing the distracter task successfully. These effects were significantly smaller for the participants who used their non-paretic arm and for the participants without stroke.

**Conclusions:**

Visual distraction decreased participants effort during a standard robot-assisted movement training task. This effect was greater for the hemiparetic arm, suggesting that the increased demands associated with controlling an affected arm make the motor system more prone to slack when distracted. Providing an alternate sensory channel for feedback, i.e., auditory feedback of tracking error, enabled the participants to simultaneously perform the tracking task and distracter task effectively. Thus, incorporating real-time auditory feedback of performance errors might improve clinical outcomes of robotic therapy systems.

## Background

Stroke is a leading cause of movement disability in the USA and Europe [1]. Repetitive and intense movement practice can help improve function after stroke [2]. However, movement therapy can be labor intensive and time consuming for therapists to provide. Robotic devices have the potential to partially automate therapy, helping individuals affected by stroke perform some forms of repetitive training in a controlled fashion, and providing feedback to stroke subjets and therapists about movement performance and training intensity.

Recognizing these potential benefits, there has been a rapid increase in development of robotic devices for rehabilitation of persons with disabilities (see reviews [3-6]). While initial results are positive, two recent reviews indicate that clinical results are still not fully satisfactory [7,8], the gain achieved using robot therapy is still small and it needs to be improved.

Currently, most robotic therapy devices physically assist the patient in performing games presented visually on a computer display. The rationale for physically assisting movement is that it provides novel sensory and soft tissue stimulation, demonstrates how better to perform a movement, and increases the motivation of the patient to engage in therapy [9]. However, an unintended and possibly negative effect of providing assistance is that subjects may reduce their effort and participation in the training. A reduction of patient effort in response to robotic assistance has been documented for both arm training [10] and gait training [11]. This reduction has been hypothesized to explain the diminished benefits of robot-assisted gait training compared to conventional gait training, although other explanations are possible such as inappropriate sensory stimulation or lack of kinematic variability in training. These are recently documented for chronic stroke patients who were ambulatory at the start of robotic training [12]. In the extreme, if a patient is passive as a robot moves his or her limbs, the effectiveness of repetitive movement training is substantially reduced [13]. But even a moderate reduction in patient effort may diminish training effectiveness.

Developing a better understanding of the brain mechanisms that control the slacking response is important for optimizing robot therapy. One view of slacking is that it is a natural consequence of the computational mechanisms that the human motor system uses to adapt to novel dynamic environments. Specifically, humans adapt to robot-generated dynamic environments in a way that appears to minimize a cost function with both error and effort terms [14]. Thus, if a robot assists in maintaining movement accuracy, in this model the motor system will systematically seek to reduce effort, as has been shown experimentally [10,15-17]. However, the instruction to the patient, psychological factors, and visual feedback [18] may also influence slacking.

The human motor system has a limited capacity to multi-task [20], therefore we hyphothesize that patients who are distracted by a secondary task might therefore reduce effort for a movement task, especially if the kinematic effects of the effort are ameliorated by robotic assistance. Consistent with this hypothesis, in a pilot study with unimpaired participants [21], we found that a relatively mild visual distracter introduced during a typical robotic therapy tracking exercise significantly increased the participants' tracking errors as well as the interaction forces against the robot. In the present study, we sought to determine whether participants with chronic stroke slacked when asked to perform a distracter task during a robot-assisted arm tracking task. We also studied whether using a secondary feedback channel, the auditory system, to inform participants of tracking error could help them better perform the tracking and distracter tasks, simultaneously, consistent with recent research that has shown that sound feedback can help subjects affected by stroke improve their tracking performance [22].

## Methods

### Subjects

Individuals with hemiparesis were included in the study if they had a chronic unilateral stroke (> 6 months), and showed some motor recovery at the affected elbow and shoulder (score > 10/42 on the Arm Motor Fugl-Meyer scale, excluding the hand and wrist components). Any subject presenting with severe spasticity (score > 4 on the modified Ash-worth spasticity scale), severe hemineglect (score ±1 on the Line Cancellation Task), ideomotor apraxia (score < 3 on either hand on the modified Alexander test) or color blindness (unable to distinguish red and green colors) was excluded. Informed consent was obtained from each subject before the evaluation session, and the UC Irvine Institutional Review Board approved the study. To determine subject's eligibility, a study member assessed motor impairment at the affected upper extremity by means of the Arm Motor Fugl-Meyer Scale (excluding the wrist and hand components; normal = 42) [23]. Spasticity at the affected upper extremity was assessed by the modified Ashworth Spasticity Scale [24] (normal = 0). Hemineglect and ideomotor apraxia were evaluated with the Line Cancellation Task (normal = 0 omissions) [25] and the ideomotor apraxia Scale (normal = 5) [26], respectively. Color blindness was assessed by presenting the subjects with two color-coded sheets (one green and one red), representing the color of the visual distracters, and asking them to name the color of each sheet. A total of 14 individuals with left hemiparesis and 5 with right hemi-paresis participated in the study. The mean age and time since stroke of the 14 participants (54% female, 46%male) were 56.3 ± 12.3 years. The mean Arm Motor Fugl-Meyer Scale was 25.9 ± 4.9, and the mean Ashworth score was 1.92 ± 0.8 and 0.86 ± 0.36 at the affected elbow and shoulder, respectively (see Table 1). No subject presented hemineglect (Line Cancellation Task score: -0.003 ± 0.001), ideomotor apraxia (5 ± 0) or color blindness. The 5 individuals with right hemiparesis (20% female, 80% male) who used their non-paretic arm for tracking had a mean age of 61.8 ± 5.0 years. Their mean Arm Motor Fugl-Meyer Scale was 36.0 ± 2.2, and the mean Ashworth score was 0.75 ± 0.5 and 0 ± 0 at the affected elbow and shoulder, respectively. We selected right hemi-paretic participants who had enough residual hand movement ability to click the mouse without difficulty. The rehabilitation robot used in this study was used in its left-handed configuration. Therefore, all participants used their left hand to perform the tracking task, yielding 14 people with stroke who participated with their paretic arm, and 5 with their non-paretic arm. We also recruited 14 participants (18% female, 82% male) with a mean age of 27 ± 7.53 years old without motor impairment, to perform the whole experiment.

**Table 1:** Subjects with left hemiparesis

**Subj**.	Age (years)	Time since stroke (months)	Gender	Arm Motor FM score (/42)	Mod. Ashworth score (/4)	
					Elbow	Shoulder
1	71	113	F	20	2	1
2	63	60	F	28	3	1
3	77	89	F	15	2	1
4	59	148	M	20	1	1
5	53	18	M	23	3	0
6	47	36	M	25	2	1
7	48	171	F	28	1	1
8	72	6	F	25	1	1
9	62	79	F	31	2	1
10	65	101	M	31	3	0
11	37	37	F	32	1	1
12	46	15	M	27	2	1
13	43	8	M	27	1	1
14	46	30	F	30	3	1

### Experimental set-up

We simulated a situation that occurs frequently during robot-assisted rehabilitation therapy in which a patient attempts to perform a visual movement tracking task, but his or her attention is perturbed by distracters appearing in the environment. In the clinic, the distracter might be other people moving or talking in the environment, the patient's own thoughts, or objects of interest in the visual field. To create a controlled experiment, we created a distracter using a secondary visual task on the computer screen.

**Figure 1:**
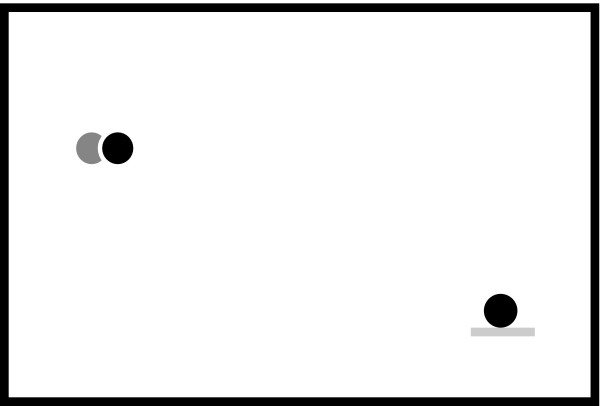
**Human Machine Interface**. Visual and audio interface used for the tracking task: Target position is represented by a red filled dot (black dot in the figure) and hand position is represented by a green filled dot (light gray dot in the figure) in a black screen (white in the figure). A visual distracter is also shown in the bottom right corner.

We designed a tracking task, similar to commonly-used robotic therapy tracking tasks, for which subjects had to follow a target on a computer screen as accurately as possible in a cyclic left-to-right movement using their affected upper extremity. Note that the movement trajectory was entirely horizontal (in the X axis), and required a left-to-right motion of about 18 inches long with a "minimum jerk" velocity profile for the target [27]. The subject's hand position (midpoint of the robot's stick handled by the subject) was represented by a green dot and the target position was represented by a red dot. The user interface was implemented using Microsoft Visual Basic .NET and OpenGL (see Figure 1). While tracking the target, the subjects were asked to click a mouse using their hand not positioned in the robot when a goal visual distracter appeared on the computer screen. The visual distracters varied randomly according to the combination of three parameters: color (red or green), position of the distracter (bottom left or right of the computer screen) and position of a yellow horizontal line (above or below the distracter); by varying these features, eight total distracters were possible. The two goal distracters were chosen from among the eight combinations, for which participants were instructed to click the mouse button, consisted of a green colored dot with a yellow line above appearing at the bottom left of the screen, or a red dot with a yellow line below appearing at the bottom right of the screen. The visual distracters were shown for 2 sec with a random time gap between 1 and 5 sec between each distracter.

**Figure 2:**
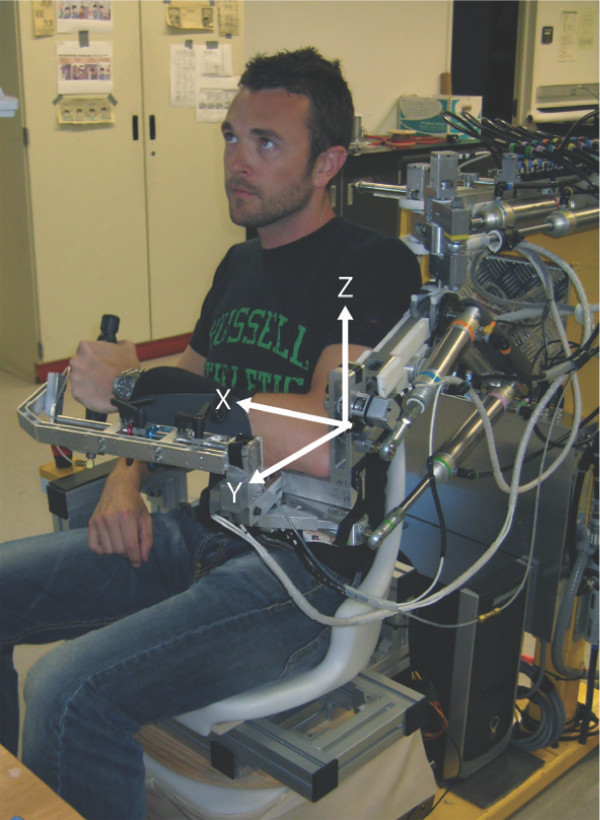
**Pneu-WREX**. Pneumatic exoskeleton [28] used to perform clinical trials.

The robot used to assist in performing the tracking task was a pneumatic exoskeleton, the Pneu-WREX [28], which has been used previously in a study of robotic therapy with over 30 participants with chronic stroke [29]. The Pneu-WREX (see Figure 2) evolved from a passive rehabilitation device called the T-WREX [30]. The Pneu-WREX is able to generate large forces within a good dynamic range (like a therapist's assistance) using nonlinear control techniques [31]. The controller used to assist the patient in moving during the experiments was an adaptive controller with a forgetting term developed previously [32]. The adaptive controller uses a measurement of tracking error to build a model of the forces needed to assist the arm in moving. The model is represented as a function of the position of the arm, using radial basis functions whose parameters are updated with a standard adaptive control law; other ways to implement the model have been developed [33]. Building a model of the forces needed to move the arm allows the robot to be made more compliant, since it no longer needs to rely solely on position feedback to decrease tracking error. Essentially, the resulting controller models the forces needed to assist the subject, as learned from tracking errors, and reduces its effort with time on an exponential basis when kinematic error is small.

For some exercises, we provided sound feedback of tracking error, developed using Microsoft DirectX9. The sound feedback was a sequence of tonal beeps, with each beep sampled at a frequency of 800*Hz *and lasting 0.1*sec*. The frequency of repetition of the tonal beeps varied proportionally to the vector magnitude of the position tracking error, with a dead zone of 1*in*. around the target. The beep was produced using either the left or the right audio channels according to the direction of error and it was provided by the speakers integrated in the monitor.

### Experimental protocol

Each subject's left upper extremity was positioned in Pneu-WREX and secured with Velcro straps (see Figure 2). Subjects were asked to complete five different tracking tasks, which were presented in random order for each subject. Overall, each task was executed by each group an equal number of times in order to avoid randomization bias:

• Task A: (the "baseline" tracking task) track the target without the visual distracter and without sound feedback

• Task B: track the target with the visual distracter and without sound feedback

• Task C: track the target with the visual distracter and with sound feedback

• Task D: track the target without the visual distracter and with sound feedback

• Task E: same as task A, but with the subject instructed to completely relax their affected upper extremity. This task provided a measurement of the arm weight of the subject, as the robot control algorithm adapted to lift the subject's passive arm to perform the tracking task, and we recorded the force the robot generated to do this.

The normalization of the force in Z axis (*F_z_*) and the position error in Z axis (Δ*Z*) were calculated for each task based of the robot assistance force provided during the task E. For example, the *F_z _*can be summarized with the following formula:

With *k *= *A, B, C, D *and *i *is the cycle during each task. The position error in Z axis is based on the following formula:

The robot assisted the subjects' tracking movement, just as in most forms of robotic-assisted therapy. Each task consisted of 20 continuous repetitions of the left-right-left movement, with each repetition lasting six seconds (total duration of each task: 120*s*). A 10-s pause was given to the participants between each task. During each task, target and hand positions, velocity, robot force and mouse button status (Tasks B and C only) were sampled at a frequency of 200*Hz *and used for analysis as well as each subject's position errors and forces for the X (left-right) and Z (up-down) axes. The Y axis (front-back) was left uncontrolled with the robot in back-drive mode in this direction.

### Data Analysis

We performed a comparison between paired groups (Shapiro-Wilk Normality Test and D'Agostino-Pearson omnibus normality test) and found that the distribution was Gaussian for data related to the force in z dimension and non-Gaussian for data related to error in z dimension. Thus we performed a parametric t-test to evaluate the robot assistance between the different tasks and non-parametric t-tests (Wilcoxon t-test) to compare the participants' position error. For the participants with stroke and healthy participants, 1 outlier was discarded in each case because the participant misunderstood the execution of the tasks. Also, we analyzed the distracter task in order to understand how the participants executed the task with/without sound feedback. The success rate was calculated as percentage of the distracter trials when the subject correctly clicked the mouse within a 2.5 second window after a goal distracter appeared.

## Results

**Figure 3:**
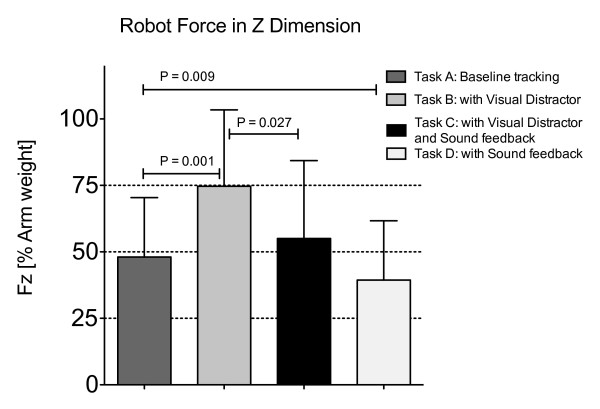
**Robot force in Z dimension**. Robot assistance force in the z (vertical) direction for participants with stroke using their paretic arms to track, relative to assistance force when the participants completely relaxed their arms in Task E.

**Figure 4:**
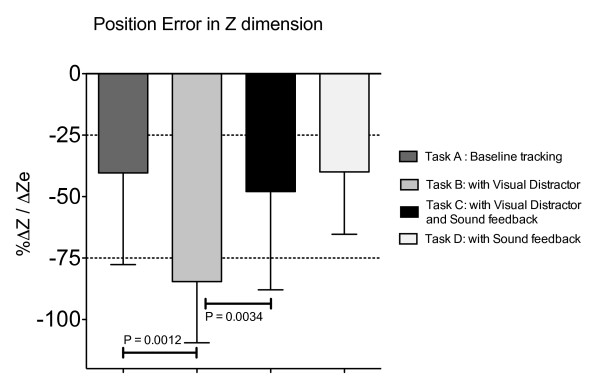
**Tracking error in Z dimension**. Position error for participants with stroke using their paretic arms to track, relative to tracking error when the participants completely relaxed their arms in Task E.

The results are presented for 13 participants with left hemiparesis secondary to a stroke, 5 participants with right hemiparesis and 13 healthy participants. For the hemiparetic arms on the baseline tracking task, the participants supported about 50% of their arm weight, with the robot adapting to provide the other 50% of support needed to lift the arm and perform the horizontal tracking task (Figure 3). Introduction of the visual distracter task caused participants to reduce their effort, as evidenced by a significant increase in the robot assistance force in the vertical (Z) direction (Figure 3, *p *= 0.001, comparison between Task A and Task B). The amount of increase was approximately 25% of arm weight; thus participants with stroke who used their impaired arm for the task reduced their force in the vertical direction by about half when performing the visual distracter task. The vertical position tracking error doubled (Figure 4, *p *= 0.0012). There were no significant increases in robot assistance force or position tracking error in the left-right (X) direction.

Again for the hemiparetic arms, sound feedback of tracking error provided during the visual distraction task significantly decreased the assistive force provided by the robot (Figure 3, *p *= 0.027) and the position error (Figure 4, *p *= 0.0034, comparison between Task B and Task C), restoring these measures close to their value during the default visual tracking task (Task A). The success rate for correctly clicking the mouse button when the distracter appeared was 65% for task B and 63% for task C.

The sound feedback also increased patient effort when no visual distracter was present. When comparing the tracking task with sound feedback (task D) to the baseline tracking task (task A), there was a significant decrease in the robot-assisted force (Figure 3, *p *= 0.009). However, no significant difference in position error was noted when comparing these two tasks (*p *> 0.05).

**Figure 5:**
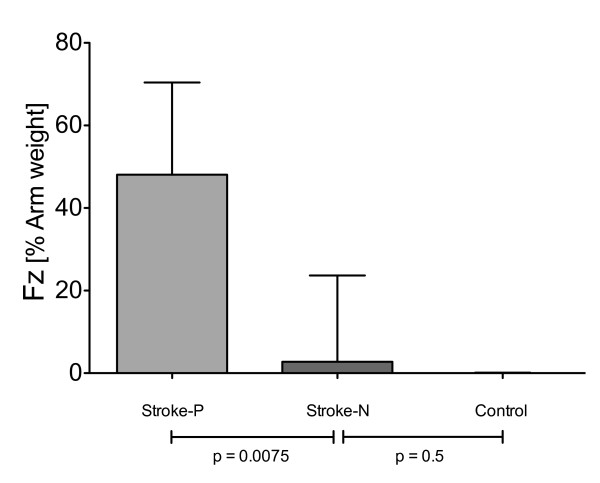
**Robot force in Z dimension during the baseline task (Task A).** (Stroke-P: stroke with paretic arm - Stroke-N: stroke with non-paretic arm - Control: subjects without impairment). Robotic assistance force in the z (vertical) direction for stroke participants using their paretic arm ("Stroke-P"), stroke participants using their nonparetic arm ("Stroke-N"), and control participants without stroke ("Control"). Task A: Baseline tracking without distractor or sound feedback.

**Figure 6:**
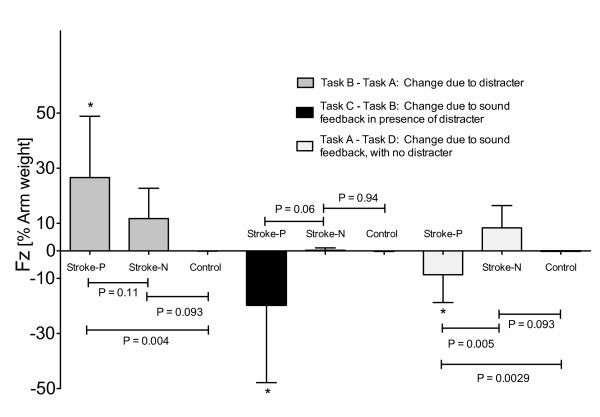
**Robot force in Z dimension between the experimental group and the control group (non-impaired arm of stroke and healthy participants)**. Change of robotic assistance force in the z (vertical) direction for stroke participants using their paretic arm ("Stroke-P"), stroke participants using their non-paretic arm ("Stroke-N"), and control participants without stroke ("Control"). Task A: Baseline tracking without distractor or sound feedback. Task B: with visual distractor. Task C: with visual distractor and sound feedback. Task D: with sound feedback and no distractor. (* = significant difference in the change of robotic assistance compare to zero assistance: in particular Task B -Task A has *p *= 0.0004, the Task C -Task B has *p *= 0.0085 and the Task A - Task D has *p *= 0.0023).

We analyzed whether the decrease in effort caused by the distracter task was related to the use of the hemiparetic arm for tracking, or whether a similar decrease was seen when a control group of 13 young, non-impaired participants and 5 participants with stroke, using their non-paretic arm, performed the tracking task. The robot adapted to provide near zero assistance when these participants used their non-paretic/non-impaired arms for the default tracking task (Figure 5). Figure 6 shows that introduction of the visual distracter caused a significant increase (*p = 0.004) in robot assistance force for hemiparetic arm, but not for the non-paretic/non-impaired arms. The size of this increase was larger for the hemiparetic arm as compared to the non-impaired arm of the young participants (*p *= 0.004), but not as compared to the non-paretic arm of the stroke participants (*p *= 0.11). The introduction of sound feedback had a greater differential impact on the force produced by the hemiparetic arm compared to the non-paretic/non-impaired arm, with or without the visual distracter (respectively: *p = 0.0085 and *p = 0.0023).

## Discussion and Conclusion

We found that participants with stroke substantially reduced their force production during a typical robot-assisted therapy tracking task, when presented with a secondary visual distractor. This effect was more pronounced when the arm used for tracking was hemiparetic. Introduction of sound feedback of tracking error allowed participants to perform the distractor task while maintaining their effort at the tracking task. We first discuss the implications of these results for robot-assisted therapy, and then discuss sound feedback with respect to robotic therapy device design.

### Distraction, attention demands, and robot-assisted therapy

An unintended consequence of robot-assisted therapy is that the patient may sometimes reduce his or her efforts toward trying to move, as has been documented for arm [10] and gait training [11]. Ironically, this reduction of effort is facilitated at least in part by the robot itself: robotic assistance preserves the desired kinematics of motion, reducing the errors that might normally keep effort levels high. Such a reduction in effort may reduce the effectiveness of training. For example, one recent study found that training with a gait robot without any feedback of effort, a training approach which had previously been documented to reduce the energy consumption of individuals affected by stroke during walking [11] compared to therapist-assisted gait training, was about half as effective as conventional gait training without robotic assistance to the legs, at least for chronic stroke subjects who were ambulatory at the study onset. Another recent study compared passive range of motion exercise of the upper extremity to EMG-triggered FES, which required effort from the patient, and found that the passive exercise was substantially less effective [13]. Comparisons of active and passive motor learning in non-impaired subjects are consistent with this finding [34-37]. If patient effort is important for promoting motor recovery, then identifying the factors that reduce effort, and designing ways to counteract these factors is important. In the present study, we found that introduction of a simple visual distracter task substantially reduced the effort of participants with chronic stroke during a standard robot-assisted therapy tracking task.

A similar reduction was not found for age-matched participants with stroke who used their non-paretic arm to reach, nor for participants without impairment. We hypothesize, first, that stroke survivors required increased attention to move their paretic arms; i.e. they have reduced automaticity for arm movement. Then, the propensity for slacking is likely tied to this increased attention requirement. These results are consistent with the finding that a secondary cognitive task reduces gait speed after stroke [38], although in that study, unlike the current one, the reduction seemed more associated with aging than the stroke per se. An interesting follow-up experiment would be to measure whether non-impaired participants slack when they make high-effort movements, to determine if the increased attention demand is related to weakness due to the stroke or the stroke itself. Attentional demand has previously been found to affect maximum force production in non-impaired subjects [39].

In this study we examined how effort changed with distraction, because we hypothesize that effort is linked to clinical outcomes. Other studies have found that short-term motor learning itself degrades in the presence of a distracter, with the degradation worse in the beginning of learning or when subjects have a motor deficit [20,36,40-44]. The present study confirms that even a simple visual task acts as an interfering influence on movement control of task after stroke, leading us to hypothesize that short term learning also would be affected by a visual distracter. This research thus suggests that it is important to remove even simple distractors from the training environment during robot-assisted movement training of people with stroke. Failure to control for distracting influences may at a minimum increase variability of results, and at worse diminish clinical benefits of robotic therapy. Another important direction for design of robot therapy is to reduce the assistance as much as possible. For example, if users of the devices experience obvious kinematic consequences when they are distracted, they may be less inclined to become distracted. In the optimization framework for modeling slacking we developed previously [14], the effects of a distractor as observed here could be accounted for by a reduction in the internal weight assigned to the effort component of the cost to minimized. In this framework, the cost function that the motor system minimizes would thus be affected by the attention demands placed on the motor system.

### Sound feedback and robot-assisted therapy

Remarkably, we found that introduction of a simple form of auditory feedback eliminated the slacking that arose from performing the secondary distracter task. Participants not only continued to perform the distracter task with a similar success rate, but increased their effort back toward their baseline levels with the aid of auditory feedback. A likely explanation is that introduction of the visual distracter task overloaded the visual-motor channel; provision of feedback through the auditory system allowed better parallel processing. Rather than acting as a confounding influence or another distracter, the sound feedback enhanced the visuo-motor control because it provided similar information [45].

An important implication of this finding is that increased attention should be paid to incorporating effective forms of auditory feedback during robot-assisted movement training. Our impression is that auditory feedback is underutilized in most robotic therapy systems, playing a role as background music or signifying only task completion, although there are attempts to use auditory feedback in a more sophisticated way (e.g. [22,46-48]. In one study, when people with chronic stroke practiced reaching with sound feedback that informed them about the deviation of their hand from the ideal path, they significantly reduced their position error after training [48]. A control group that did the same exercise without feedback did not improve its performance. In another study, a virtual reality training system that incorporated sound feedback of reach position and speed helped subjects with traumatic brain injury improve their reaching ability [49]. Another study found that lower extremity training of individuals with chronic hemiparesis using a robotic device coupled with Virtual Reality (including visual and audio feedback) improved walking ability in the laboratory and the community better than robot training alone [50].

These studies suggest that incorporation of augmented feedback can improve not only performance but also long-term motor learning after stroke. In the present study, we only demonstrated that auditory feedback improves short-term performance, measured by force output and tracking error. Future studies are needed to determine how providing auditory feedback of error can best improve learning of arm movement after stroke. We hypothesize that auditory feedback can serve to keep the subjects effort level elevated, as demonstrated here, which should improve use-dependent plasticity by reducing passivity. However, there is a possibility that subjects could come to rely on the auditory feedback to drive their performance, reducing transfer to real-life arm movements in which auditory feedback is not available. Thus, in testing the long-term effect of auditory feedback, in may be important to fade the feedback, or to provide it only intermittently, in order to reduce any possible growing dependence on it. Further, challenging the patient by intermittently providing a distracting environment with and without the aid of auditory feedback to overcome that distraction may be an appropriate way to allow people to learn to move well in the presence of distractors.

Another recent study found that the effect of sound feedback during reaching after chronic stroke depended on the hemisphere that was damaged by the stroke [22]. In this study, participants heard a buzzing sound similar to the sound of a fly, with the volume of the buzz increasing with proximity to a reach target, and in some cases, the spatial balance of stereo sound was also altered by the orientation of the hand with respect to the target. Such sound feedback improved abnormal curvature in participants with right hemisphere damage (i.e. participants who were left hemiparetic, like the ones in our study), and degraded curvature, peak velocity, and smoothness in participants with left hemisphere damage [22]. Robertson suggested that this result might be explained by either a difference in processing of auditory information, possibly due to receptive aphasia associated with left hemisphere damage, or to the fact that each hemisphere has a different role in movement control.

In the current study, we used a small sample of people with left hemiparesis for convenience: the robot was setup for left-handed use, and switching it was cumbersome. This choice may have been fortuitous, as the Robertson study suggests that people with left hemiparesis benefit more from sound feed-back. Further investigation is needed to understand if the sound feedback provided during a distraction task could be helpful also for right-hemiparetic subjects. Another factor affecting generalizability of the current results is that the participants recruited presented a narrow range of impairments at the affected upper extremity (Fugl-Meyer score range 15-32). In addition, the study excluded individuals presenting severe impairments at the affected upper extremity, which represent up to 30% of stroke survivors [51]. Future studies should look also at the impact of auditory feedback on a broader spectrum of level of impairment after stroke. Finally, upcoming research should also examine how auditory feedback can best be crafted to improve learning and motor recovery.

## Competing interests

The authors declare that they have no competing interests.

## Authors' contributions

RS designed and developed the multi-feedback interface, ran the study (design of experiments and ran clinical trials), performed the statistical analysis and drafted the manuscript. MH helped during the clinical trials, carried out to the recruitment of subjects and assessed the medical trials. DJR and GR contributed concepts, edited and revised the manuscript. All authors read, edited and approved the manuscript.
